# Effect of Ku70 expression on radiosensitivity in renal carcinoma 786-O cells

**DOI:** 10.1186/1475-2867-14-44

**Published:** 2014-05-26

**Authors:** Defeng Qi, Yuan Hu, Yangde Zhang, Tao Peng, Weidong Ji

**Affiliations:** 1 National Hepatobiliary and Enteric Surgery Research Center, Centeral South University, Changsha 410008, China; 2 Department of Urology, Minimally Invasive Surgery Center, Guangdong Provincial Key Laboratory of Urology, The First Affiliated Hospital of Guangzhou Medical University, Guangzhou 510230, China

**Keywords:** Renal cell carcinoma, Radiosensitivity, Ku70, Cell apoptosis

## Abstract

**Background:**

Radiotherapy plays an important role in cancer therapy. However, the radioresistance of some human cancers, particularly renal carcinoma, often results in radiotherapy failure. The Ku protein is essential for the repair of a majority of DNA double-strand breaks in mammalian cells, but effect of Ku70 expression on radiosensitivity in renal carcinoma is unclear. Here, we investigate the impact of Ku70 on radiosensitivity in renal carcinoma cells through regulating the expression of Ku70.

**Methods:**

The stable overexpression of Ku70 or suppression of Ku70 in renal carcinoma cell line (786-O) was generated by retrovirus-mediated Ku70 cDNA or shRNA targeting Ku70. Ku70 expression was determined by RT-PCR and Western blot analysis, the apoptosis of the stable cells was assayed with flow cytometry and TUNEL assay and the effect of radiation on the livability of stable cells was assessed by MTT assay.

**Results:**

Up-regulation of Ku70 expression of 786-O cells could inhibit cell apoptosis and reduce susceptibility to radiation. On the contrary, 786-O cells with suppression of Ku70 expression could induce cell apoptosis and significantly enhance the sensitivity to radiation.

**Conclusions:**

These findings indicated that Ku70 might play an important role in radioresistance of renal carcinoma, and inhibition of Ku70 can increase the radiosensitivity of 786-O cells by enhancing apoptosis, suggesting down-regulation of Ku70 expression combined with radiotherapy will be a potential strategy for renal cell carcinoma therapy.

## Background

Renal cell carcinoma (RCC) represents 2-3% of all cancers [1], with the highest incidence occurring in Western countries. During the last two decades, there has been an annual increase of about 2% in incidence both worldwide and in Europe, though in Denmark and Sweden a continuing decrease has been observed [2]. Renal cell carcinoma is the commonest solid lesion within the kidney and accounts for approximately 90% of all kidney malignancies. To date, treating RCC is still a very challenging task because there is no effective strategy in treating the late stages of this disease, so it is usually followed by a poor prognosis. Under the current circumstances, it is essential to develop more effective therapeutic strategies for RCC.

DNA double-strand breaks (DSBs) caused by endogenous (byproducts of cellular metabolism and replication associated errors) and exogenous (ionizing radiation and chemotherapeutic drugs) agents, are the most lethal damage among the different kind of DNA damages as unrepaired DSBs can result in genomic instability, cell death and tumorigenesis [3-5]. It can be repaired via two major pathways: homologous recombination (HR) and nonhomologous end joining (NHEJ). The NHEJ pathway, however, is regarded as the major pathway for the repair of radiation induced DSBs in mammalian cells [6-10]. One of the main participants in this pathway is the DNA-dependent protein kinase (DNA-PK) that consists of a large catalytic subunit, DNA-PKcs, and a heterodimeric protein named Ku, which is a highly stable protein complex consisting of a 70 kDa and a 86 kDa polypeptide, better known as Ku70 and Ku80 [11-15]. NHEJ is initiated by the DNA repair protein Ku, which recognizes DSBs and recruits additional pathway components to process and repair the damage. Therefore, Ku plays a crucial role for DSBs repair.

The role of DNA-PK complex in the radiosensitivity of human cells is still being investigated. Of significance for radiation oncology is the evidence that defect in or absence of Ku70, Ku80, or DNA-PKcs subunit results in deficiencies in DNA-DSB repair, leading to hypersensitivity to ionizing radiation [16-18]. Given the crucial role of the DNA-PK complex in determining the response of cells to radiation, targeting the components of this complex represents an appealing opportunity to increase the radiosensitivity of mammalian cells [19,20].

The DNA repair protein Ku70 is a key factor for radioresistance, and previous reports have demonstrated that suppression of Ku70 using the antisense nucleic acid strategy increases radiosensitivity [19,21]. However, the role of Ku70 as targets for radiation in RCC cells has not been addressed. In the present studies, to investigate the association of Ku70 expression with radiosensitivity of RCC cells, we compared the radiosensitivity of 786-O cells up-regulating and down-regulating Ku70 with that of their control groups. Our date shows that reducing cellular Ku70 level in 786-O cells increased more radiosensitive, likely in part through inducing cell apoptosis. While increasing cellular Ku70 level could make cells more radioresistant. These results support that suppressing Ku70 expression may be a potential therapeutic strategy to enhance the sensitivity of RCC cells to radiation.

## Results

### Ku70 expression in stable cell lines

**Figure 1:**
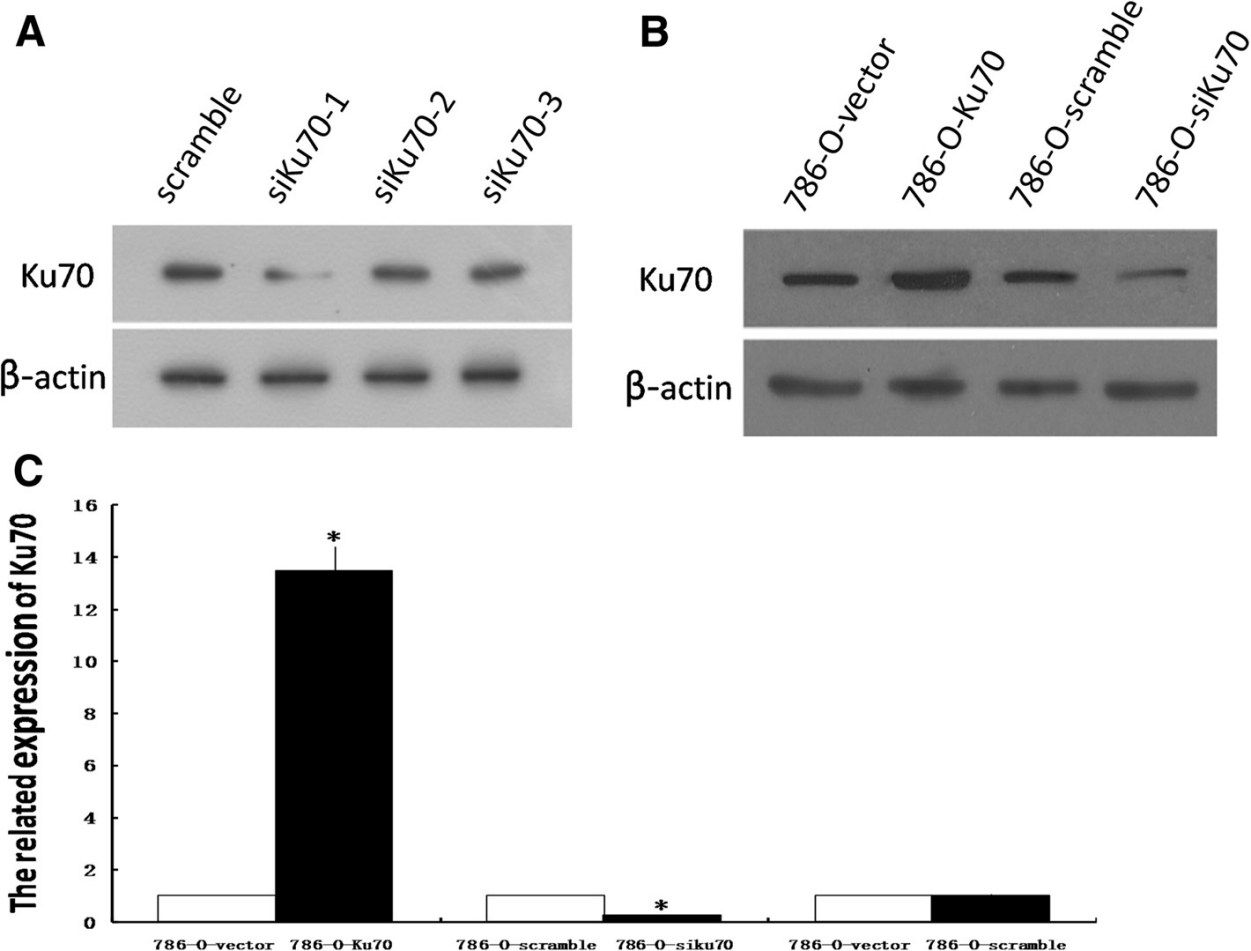
**Ku70 expression was measured in the stable 786-O cell lines. A**: Effect of various siRNAs on Ku70 expression was analysed by Western blot in 786-O cells. **B**: Ku70 mRNA expression was detected by RT-PCR in the stable 786-O cell lines. **C**: Ku70 protein expression was detected by Western blot in the stable 786-O cell lines. *P < 0.05 is considered significant.

In order to construct the pBaBb-puro-Ku70 vector containing a Ku70 cDNA and the pSUPERretro-puro-siKu70 vector, a DNA fragment encoding Ku70 was amplified by PCR from human genomic DNA and three pairs of effective RNA interference sequences (siKu70-1, siKu70-2 and siKu70-3) to target Ku70 gene were designed and synthesized. To determine the effect of various siRNAs on Ku70 protein expression, we measured the amount of Ku70 by Western blot analysis. Of the three Ku70 siRNAs, siKu70-1 was the most effective in suppressing the expression of Ku70 (Figure 1A). Therefore, the recombinant plasmid vector containing siKu70-1 was screened out for establishing stably knock-down 786-O cell line.Ku70 expression in stable cell lines was determined by RT-PCR and Western blot analysis. Compared with stable 786-O control cells, the expression of Ku70 mRNA was increased by more than 13-fold in 786-O-Ku70 cells, and reduced by 76% in 786-O-siKu70 cells (Figure 1B). Additionally, 786-O-vector cells and 786-O-scramble cells showed no significant change of Ku70 expression (Figure 1B and 1C). The results indicate the stable 786-O cell lines with overexpression or downexpression of Ku70 were successfully generated by retrovirus-mediated method.

### Effect of Ku70 expression on apoptosis in 786-O cells

**Figure 2:**
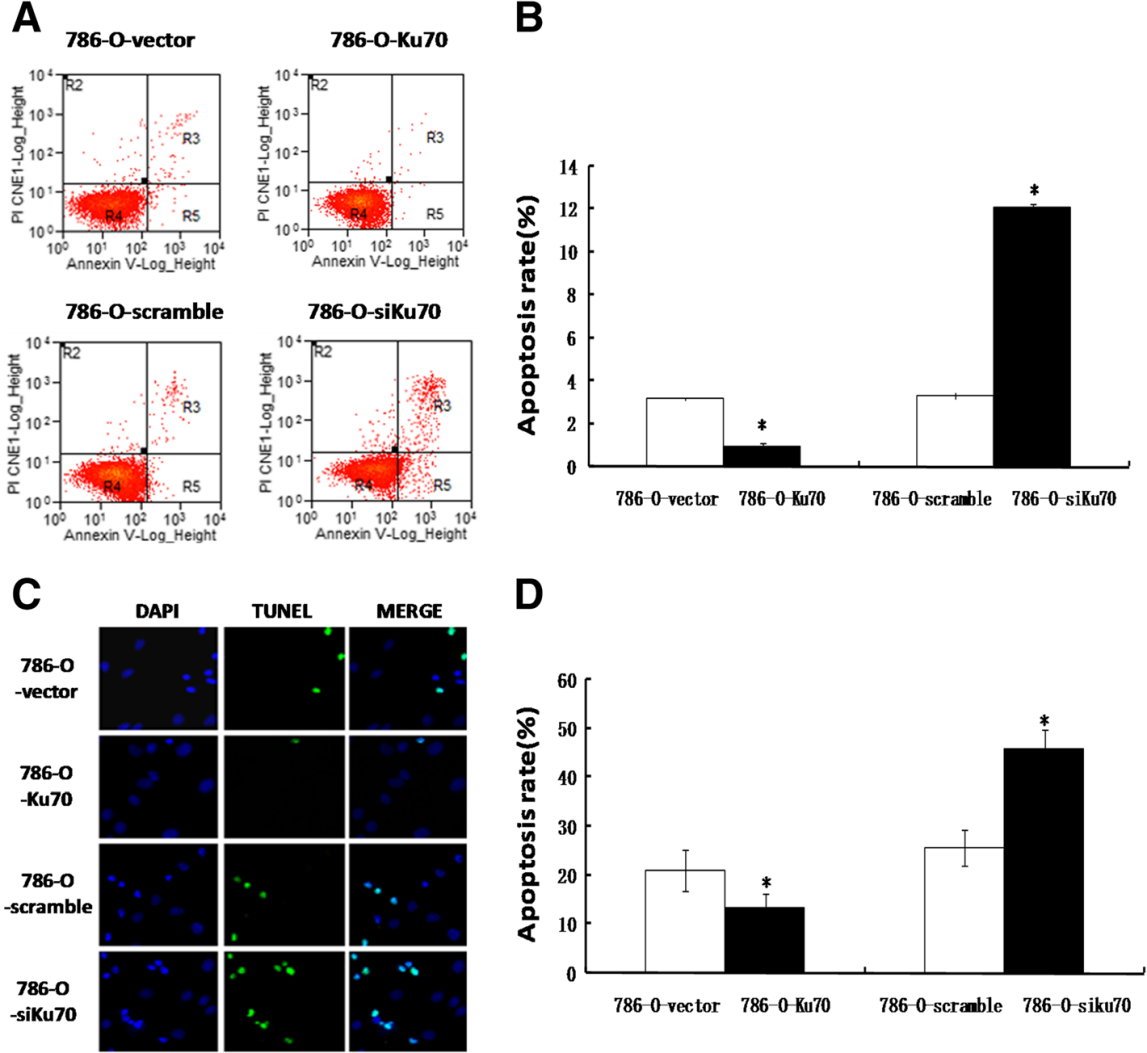
**Ku70 suppress the apoptosis of 786-O cells. A**: Images showing flow cytometric analysis of apoptosis. **B**: Images showing TUNEL assay of apoptosis. **C** and **D**: The histogram shows the results from A and B (%). *P < 0.05. Results are representative of three independent experiments.

To investigate whether the amount of Ku70 could affect the apoptosis of RCC cells, flow cytometry and TUNEL assay were performed to analyze the apoptosis of the stable 786-O cell lines.The flow cytometry analysis indicated that the apoptosis rate of 786-O-Ku70 cells (0.97 ± 0.12%) was significantly reduced in comparison with those of 786-O-vector cells (3.17 ± 0.06%). However, compared with the 786-O-scramble cells (3.33 ± 0.15%), 786-O-siKu70 cells (12.07 ± 0.15%) was significantly increased (Figure 2A and 2C).The similar results were found by TUNEL assay. As shown in Figure 2B and 2D, the apoptosis rate of cells was lower in 786-O-Ku70 cells (13.43 ± 2.89%) than in 786-O-vector cells (21.03 ± 4.18%), and the apoptosis rate of 786-O-siKu70 cells (45.70 ± 3.99%) was significantly higher than 786-O-scramble cells (25.56 ± 3.70%). All of the above results of flow cytometry analysis and TUNEL assay had statistical significance (P < 0.05). These results showed that Ku70 can suppress the apoptosis of 786-O cells.

### Effect of Ku70 expression on radiosensitivity in 786-O cells

**Figure 3:**
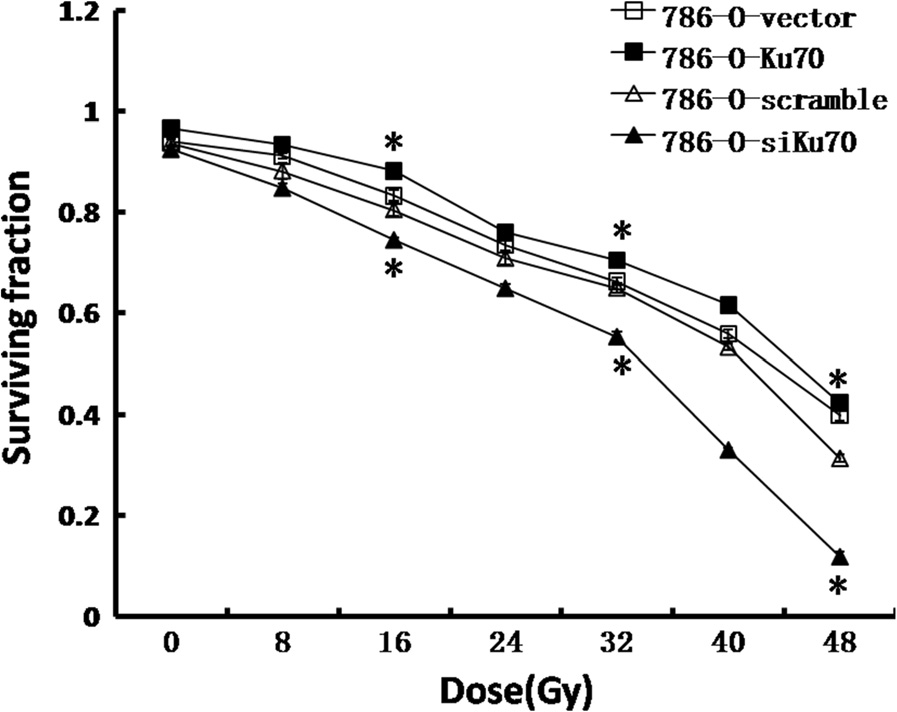
**Response of 786-O-vector, 786-O-Ku70, 786-O-scramble and 786-O-siKu70 cells to γ radiation.** Cells were exposed to γ radiation and survival assessed by MTT assay. *P < 0.05. Each date point is the mean of three independent experiments.

In order to determine the correlation between Ku70 expression and radiation response, we exposed the stable 786-O cell lines (786-O-Ku70, 786-O-siKu70, 786-O-vector and 786-O-scramble) to γ radiation, and assessed the cellular radiosensitivity using MTT assay (Figure 3). Compared to the 786-O-vector cells, the survival fractions of 786-O-Ku70 cells were much higher at each point, and at 16Gy, 32Gy and 48Gy, the survival fraction of 786-O-Ku70 cells was increased by 4.9%, 3.8% and 2.3%, respectively. Conversely, the survival fractions of 786-O-siKu70 cells were significantly lower than the 786-O-scramble group at each point, and at 16Gy, 32Gy and 48Gy, the survival fraction of 786-O-siKu70 cells was decreased by 6.0%, 9.7% and 19.6%, respectively. Together, the results indicated that upregulating Ku70 expression can reduce the radiosensitivity of 786-O cells. On the contrary, RNAi-mediated Ku70 knockdown enhances radiosensitivity of 786-O cells.

## Discussion

RCC has traditionally been considered intrinsically radioresistant. Susskind et al. also reported that radiation therapy is ineffective in treating this disease because the cells are relatively radioresistant, and high levels of the metastasis-promoting genes matrix metalloproteinases have also been correlated with ionizing radiation [22]. To date, the specific molecular mechanisms responsible for such radioresistance have not been elucidated. However, in the past several years, some researchers have thought up new strategies to enhance the sensitivity of RCC cells to radiation. Smyth et al. found that a dual role for TIMP-1 overexpression in renal tumour cell line as radiosensitizing agents [23]. Lei et al. also showed that knockdown of survivin enhance in vitro radiosensitivity of RCC cells [24]. Additionally, Palayoor and his study groups reported that C2 and C2VHL RCC cells were treated with 1.5 mM ibuprofen and exposed to radiation, ibuprofen enhances the radiosensitivity of both cell lines [25]. Despite this, the role of Ku70 as targets for radiation in RCC cells was not addressed in those studies.

Ku70, as a important role in the repair of radiation induced DSBs, has been a target for exploring the combination of gene and radiation therapy with the goal of using gene therapy approaches to increase the radiosensitivity of various tumors. Previous studies indicated that Ku70-deficient embryonic stem cells and bone marrow cells from Ku70 knockout mice (Ku70 −/−) show an increased sensitivity to ionizing radiation [18,26]. Ayene et al. also reported that transfection of HeLa and HCT116 cancer cells with Ku70 siRNA significantly enhance the response to γ radiation [27]. Li et al. showed that adenovirus-mediated, heat-activated antisense Ku70 expression can modulate Ku70 protein level and radiosensitize mouse fibrosarcoma cells in vitro and in vivo [19]. Furthermore, up-regulation of Ku70 expression can enhance the radioresistance of human cancer cells. For example, Otomo et al. reported that upregulated levels of Ku70 expression are significantly associated with radioresistance of glioblastoma cells [28]. Based on the above research achievements, we provide our idea for using of Ku70 as a target gene to sensitize RCC cells to ionizing radiation.

In the present study, we investigated the role of the Ku70 in the response of human renal carcinoma 786-O cells after exposure to 0-48 Gy of γ radiation. To this end, the stable overexpression of Ku70 or suppression of Ku70 in renal carcinoma cell lines (786-O) were generated by retrovirus-mediated Ku70 cDNA or shRNA targeting Ku70, respectively, and cellular radiosensitivity was assessed. The results showed that up-regulation of Ku70 expression of 786-O cells could reduce the radiosensitivity to radiation; on the contrary, 786-O cells with suppression of Ku70 expression could result in increased radiosensitivity. These date suggest that the expression of Ku70 correlates with radiosensitivity in renal carcinoma cells.

It is well known that Ku sustains cell survival after DNA damage due to exposure to ionizing radiation. Our results not only demonstrated the ability of Ku to repair DNA damage, but also showed that inhibition of apoptosis is another function of Ku70 to affect the radiosensitivity. We performed the flow cytometry and TUNEL assay to detect the apoptosis of the stable 786-O cell lines. We found Ku70 can suppress the apoptosis of 786-O cells. Consistent with these results, Ajmani demonstrated inhibition of Ku70 expression resulted in the induction of apoptosis in human promyelocytic leukaemia HL-60 cells and activated human peripheral blood lymphocytes [29]. Sawada also reported that Ku70 plays a pivotal role in inhibition of apoptosis [30,31]. Therefore, we speculate that down-regulation of Ku70 enhance the sensitivity to radiation, likely in part through inducing cell apoptosis in renal carcinoma cells.

To our knowledge, this study is the first to provide direct evidence that the expression of Ku70 correlates with radiosensitivity in RCC cells, although many studies have arrived at the similar results in cell lines derived from other different types of tumour. Since our present study was designed to detect the link between Ku70 expression and radiosensitivity in RCC cells, we tentatively selected one kind of RCC cell lines to conduct this research. In order to achieve more convincing results and evaluate the effectiveness of this method, other cell lines of RCC and normal renal cell lines should be considered in further research.

## Conclusions

In conclusion, the expression of Ku70 correlates with radiosensitivity in RCC cells and suppression of Ku70 expression can significantly enhance the sensitivity of RCC cells to radiation, suggesting that down-regulation of Ku70 expression combined with radiotherapy will be a potential strategy for RCC therapy.

## Materials and methods

### Cell culture

Human renal carcinoma cell line (786-O) was obtained from Keygen company (Nanjing, China) and cultured in RPMI 1640 medium (Gibco, USA) supplemented with 10% fetal bovine serum (Gibco, USA) at 37°C with 5% CO_2_ and humidity-saturated incubator.

### Construction of recombinant retroviral vectors and establishment of stable human renal carcinoma 786-O cell lines

To construct a retroviral vector expressing Ku70, a DNA fragment encoding Ku70 was amplified by PCR from human genomic DNA and the cloning primer was designed as Additional file 1: Table S1. The PCR product was digested with the restriction enzymes BamH I and Sal I, and inserted into the pBaBb-puro vector. The recombinant vector was designated as pBaBb-puro-Ku70 and the correction of the inserted fragment was verified by enzymatic treatment and DNA sequencing. We also designed and synthesized three pairs of effective RNA interference (RNAi) sequences (Additional file 1: Table S2) to target Ku70 gene. Then each sequence was annealed to form double-stranded DNA and connected to pSUPERretro-puro vector digested by Bgl II and Hind III. The recombinant vectors were designated as pSUPERretro-puro-siKu70 and identified by sequencing. At the same time, we also constructed the corresponding control vectors named pBaBb-puro-vector and pSUPERretro-puro-scramble. After the Ku70 siRNA target sequence with the strongest silencing effect was screened, all of the vectors were co-transfected in 293FT cells. Generated virus particles subsequently infected 786-O cells, the positive clones were obtained following puromycin selection. The stable cell lines achieved were correspondingly designated as 786-O-Ku70, 786-O-siKu70, 786-O-vector and 786-O-scramble.

### Western blot analysis

Cultured cells were collected and washed twice with 1 ml of PBS. After cracked with total protein lysis buffer, the cells were cooled on ice for 10 min and collected in a centrifuge tube. 50 μl of protein sample were mixed with 50 μl of SDS loading buffer and incubated for 5 min at 100°C. Then the samples were run on a SDS-PAGE gel, after electrophoresis, the proteins were transferred onto PVDF membrane and detected by immunolabelling with primary and secondary antibodies. In this experiment, we made the β-actin as the internal reference.

### Real-time quantitative PCR

Total RNA was extracted using the Trizol reagent according to the instructions. The RNA purity and concentration were determined by the UV spectrophotometer. CDNA was reversibly transcribed from the extracted total RNA using an MMLV reagent kit (TaKaRa, Japan) and the primers were designed as Additional file 1: Table S1. PCR was then carried out as follows: denaturing at 95°C for 20 s, 40 cycles of 10 s at 95°C, 20 s at 58°C and 30 s at 72°C.

### Flow cytometry

The stable 786-O cell lines were harvested by centrifugation for 3 min at 1000 rpm and were resuspended in binding buffer. Aliquots containing 1 × 10^5^ cells in 190 μl of buffer were stained with 10 μl of PI solution and with 5 μl of Annexin V-FITC (eBioscience, USA) for 10 min at room temperature. Then Flow cytometric analysis was performed using a flow cytometer (BD, USA) to detect the cell apoptosis.

### TUNEL assay

TUNEL assay was also performed to detect the apoptosis of the stable 786-O cell lines. Briefly, the cells were smeared on slides and fixed with 4% paraformaldehyde at room temperature for 30 min. Then the slides were incubated with 0.1% Triton X-100 for 10 min, rinsed with PBS, incubated with 3% H_2_O_2_ to block the endogenous peroxidase activity, and rinsed with PBS. After that, the slides were TUNEL stained using a apoptosis detection kit (Keygen, China) in accordance with the manufacturer’s instructions. The TUNEL-stained slides were observed under a fluorescence microscope (Olympus, Japan).

### Radiation treatment and MTT assay

The stable 786-O cells were plated in a 96-well culture plate at an initial density of 2 × 10^3^ cells/well and then exposed to 0-48 Gy of a ^137^Cr source in a γ-irradiator. Cell viability was determined by the MTT assay immediately after radiation treatment. In brief, 20 μl of MTT solution was added into each well and incubated for 4 hours at 37°C in a humid, 5% CO_2_ atmosphere. Then, the media was removed, and cells dissolved in 150 μl of DMSO. Finally, absorbance was recorded at 490 nm using the enzyme-linked immunosorbent assay (ELISA) Reader (Bio-RAD, USA).

### Statistical analysis

All of the experiments were replicated three times. Statistical analysis was performed by independent-samples *T* Test using software SPSS 13.0. P < 0.05 was considered to be statistically significant.

## Competing interests

The authors declare that they have no competing interests.

## Authors’ contributions

QDF and JWD conceived and designed the study. HY and PT performed the experiments. ZYD assisted in the laboratory studies. HY analyzed the data and wrote the paper. All authors read and approved the final manuscript.

## Supplementary Material

Additional file 1 Primers and RNAi sequences used in this study. Click here for file
