# Supplementary material for: Effect of Ku70 expression on radiosensitivity in renal carcinoma 786-O cells
Source: Cancer Cell Int. 2014 May 26;14:44. doi: 10.1186/1475-2867-14-44 (PMC4047436; doi:10.1186/1475-2867-14-44)
Supplement: Additional file 1 — Primers and RNAi sequences used in this study. [file 1475-2867-14-44-S1.doc]

**Additional file 1**

Table S1. Primers used in this study

| Primer Name | Primer sequence（5′------3′） | |
| --- | --- | --- |
| **RT-Q-PCR primers** |  | |
| Ku70 | F: CGAGATACAGGCATCTTCCTTG | R: TCCGCAACAGGTCTTCTAGC |
| β-actin | F: TGGCACCCAGCACAATGAA | R: CTAAGTCATAGTCCGCCTAGAAGCA |
| **Cloning primers** |  |  |
| Ku70 | F: CGGGATCCATGTCAGGGTGGGAGTCATATT | R:ACGCGTCGACTCAGTCCTGGAAGTGCTTGG |
|  |  |  |

Table S2. RNAi sequences used in this study

| sequences Name | RNAi sequence（5′------3′） | |
| --- | --- | --- |
| siKu70- 1 | F: GATCCCCGTCAGGGTGGGAGTCATATTA TTCAAGAGATAATATGACTCCCACCCTGAC TTTTT | R: AGCTTAAAAAGTCAGGGTGGGAGTCATATTA TCTCTTGAATAATATGACTCCCACCCTGACGGG |
| siKu70-2 | F: GATCCCCGCTTCGCTTCACATACAGA TTCAAGAGATCTGTATGTGAAGCGAAGC TTTTTA | R: AGCTTAAAAAGCTTCGCTTCACATACAGA TCTCTTGAATCTGTATGTGAAGCGAAGCGGG |
| siKu70-3 | F: GATCCCCGCAGTGGACCTGACATTGC TTCAAGAGAGCAATGTCAGGTCCACTGC TTTTTA | R: AGCTTAAAAAGCAGTGGACCTGACATTGC TCTCTTGAAGCAATGTCAGGTCCACTGCGGG |
| scramble | F: GATCCCCGCCAGCTTAGCACTGACTC TTCAAGAGAGAGTCAGTGCTAAGCTGGC TTTTTA | R: AGCTTAAAAAGCCAGCTTAGCACTGACTC TCTCTTGAAGAGTCAGTGCTAAGCTGGCGGG |
